# *Ilex rotunda Thunb* Protects Against Dextran Sulfate Sodium-Induced Ulcerative Colitis in Mice by Restoring the Intestinal Mucosal Barrier and Modulating the Oncostatin M/Oncostatin M Receptor Pathway

**DOI:** 10.3389/fphar.2022.819826

**Published:** 2022-05-13

**Authors:** Yao Li, Xu Yang, Jia-ni Yuan, Rui Lin, Yun-yuan Tian, Yu-xin Li, Yan Zhang, Xu-fang Wang, Yan-hua Xie, Si-wang Wang, Xiao-hui Zheng

**Affiliations:** ^1^ The College of Life Sciences, Northwest University, Xi’an, China; ^2^ Air Force Hospital of Western Theater Command, Chengdu, China; ^3^ Department of Pharmacy, Xijing Hospital, Xi’an, China; ^4^ Department of Chinese Materia Medica and Natural Medicines, Air Force Medical University, Xi’an, China

**Keywords:** *Ilex rotunda thunb*, ulcerative colitis, inflammation, intestinal mucosal barrier, OSM/OSMR pathway

## Abstract

*Ilex rotunda Thunb* (IR) is a traditional Chinese medicine used for the clinical treatment of gastric ulcers and duodenal ulcers; however, the effect of IR on ulcerative colitis (UC) and its underlying mechanism remains unclear. This study investigated the therapeutic effect of IR on UC mice induced by dextran sulfate sodium (DSS) as well as the potential underlying mechanism. The main components of IR were analyzed by ultra-performance liquid chromatography-quadrupole time-of-flight mass spectrometry. Then we established a model of UC mice by administering 2.0% DSS for 7 days followed by 2 weeks of tap water for three cycles and administered IR. On day 56, the disease activity index (DAI), colon length, pathological changes, and inflammatory response of the colon tissue of mice were assessed. The oxidative stress and apoptosis of colon tissue were detected, and the integrity of the intestinal mucosal barrier was evaluated to assess the effect of IR. Furthermore, the relationship between oncostatin M (OSM) and its receptor (OSMR) in addition to the IR treatment of UC were evaluated using a mouse model and Caco2 cell model. The results showed that IR significantly alleviated the symptoms of UC including rescuing the shortened colon length; reducing DAI scores, serum myeloperoxidase and lipopolysaccharide levels, pathological damage, inflammatory cell infiltration and mRNA levels of interleukin one beta, tumor necrosis factor alpha, and interleukin six in colon tissue; alleviating oxidative stress and apoptosis by decreasing kelch-like ECH-associated protein 1 expression and increasing nuclear factor-erythroid factor 2-related factor 2 and heme oxygenase-1 protein expression; and promoting the regeneration of epithelial cells. IR also promoted the restoration of the intestinal mucosal barrier and modulated the OSM/OSMR pathway to alleviate UC. It was found that IR exerted therapeutic effects on UC by restoring the intestinal mucosal barrier and regulating the OSM/OSMR pathway.

## Introduction

Ulcerative colitis (UC) is an inflammatory bowel disease (IBD) with a complex etiology involving the interaction of genetic susceptibility, environmental factors, intestinal microbial disorders, and dysregulated immune responses (Adams and Bornemann, 2013). The occurrence of UC affects the quality of patients’ life and health. More than 80% of patients experience fatigue, weakness, and exhaustion during disease episodes; more than half of patients have their work and studies affected by IBD; and even the fertility of some female patients is affected (Kim et al., 2017; Clark-Snustad et al., 2020; Truta, 2021). Epidemiological investigation has suggested that the incidence of IBD is increasing in countries such as Africa, Asia, and South America with the economic development and changing lifestyles (Ng et al., 2017). The annual percentage change of UC in Brazil and Taiwan has increased by 14.9 and 4.8%, respectively (Ng et al., 2017). The average incidence of IBD was 14,000 per 100,000 in Asia in 2011, and the incidence of UC was twice that of Crohn’s disease (Du and Ha, 2020). Although the incidence of IBD is stabilizing in Western countries, the burden remains high because the prevalence still exceeds 0.3% (Sairenji et al., 2017). Currently, the medications for UC include 5-aminosalicylic acid (5-ASA), hormones, immunosuppressants, and biologics. 5-ASA drugs are the first-line treatment for mild to moderate UC (de Chambrun et al., 2018). Furthermore, corticosteroids are an option for patients who fail to achieve remission with 5-ASA. Anti-tumor necrosis factor alpha (TNF-α) drugs such as infliximab are considered for patients who do not respond to corticosteroid therapy (Effinger et al., 2020). Despite considerable advances in the recognition and treatment of UC, the use of 5-ASA drugs such as mesalazine is still accompanied by a series of adverse events including inflammatory reactions, pancreatitis, cardiotoxicity, hepatotoxicity, musculoskeletal complaints, respiratory symptoms, nephropathies and sexual dysfunction (Sehgal, et al., 2018). The incidence of adverse reactions of biological agents such as infliximab is 10.5%, and more than 40% of patients do not respond to infliximab treatment in clinical practice (Danese et al., 2015; Danese et al., 2019). Thus, there is a need for research and innovation into preventive and therapeutic drugs for UC.

The dried bark of *Ilex rotunda Thunb* (IR) of the family *Aquifoliaceae* with the name “Jiubiying” has been included in the “Chinese Pharmacopoeia,” and has heat-clearing and detoxifying effects, removes dampness, and relieves pain (Wang et al., 2014). It is used for the treatment of damp-heat diarrhea, abdominal pain, and bloating in traditional clinical applications. The chemical components of IR are triterpenes and their glycosides, steroids, and aromatics (Kim et al., 2012). And, triterpenes and their glycosides and aromatic compounds have the highest content in IR (Kim et al., 2012). The “Chinese Pharmacopoeia,” of 2020 specifies pedunculoside and syringin as quality-controlled components of IR, and requires their content to be ≥1.0% and ≥4.5%, respectively. Pedunculoside can prevent collagen-induced arthritis and dextran sulfate sodium (DSS)-induced acute UC (Ma et al., 2019; Liu et al., 2020). Syringin can inhibit LPS- or DSS-induced acute UC by inhibiting the activation of nuclear factor kappa B (NF-κB) and nuclear factor-erythroid factor 2-related factor 2 (Nrf2) (Zhang et al., 2020). Furthermore, previous studies have shown that rotundic acid derived from IR can treat non-alcoholic steatohepatitis, lipopolysaccharide (LPS)-induced lung damage, and colitis-related cancer (Han et al., 2019; Li X. X. et al., 2021; Liu et al., 2021). In addition, the triterpenoids in IR have anti-platelet aggregation effects (Yang et al., 2018); however, the preventive and therapeutic effects of IR and its underlying mechanism of action in chronic UC mice remain unclear. Therefore, in this study, we investigated the therapeutic effects of IR in mice with chronic UC, and illustrated the mechanisms of action related to restoration of intestinal mucosal barrier and regulation of the OSM/OSMR pathway.

## Materials and Methods

### Extract of *Ilex rotunda Thunb*

The raw herb of IR was purchased from Xi’an Traditional Chinese Herbal Medicine Co. Ltd. (Batch No: 20200118; Xi’an, China) and was identified by associate chief pharmacist Ling-bian Sun. IR samples (500 g) were extracted twice with water, first with 4 L of water for 2 h and then with 3 L of water for 1 h. Then the two filtrates were combined and concentrated and dried with a high-speed centrifugal spray dryer (HSD-8; Shanghai Universal Pharmaceutical Machinery Co., Ltd., Shanghai, China).

### Characterization of *Ilex rotunda Thunb* by Ultra-Performance Liquid Chromatography-Quadrupole Time-of-Flight Mass Spectrometry

The IR samples were separated and analyzed using the Waters I-Class VION IMS QTOF system coupled with the ACQUITY UPLC BEH C_18_ column (1.7 µm, 2.1 × 50 mm; Waters Co., Wilmslow, UK). The column temperature was 35°C, the flow rate was 0.4 ml/min, and the injection volume was 2 µl. Ultrapure water with 0.01% formic acid (A) and methanol (B) served as the mobile phase, and the gradient elution program was as follows: 0–3 min, 5–24% B; 3–5 min, 24–52% B; 5–10 min, 52–66% B; 10–15 min, 66–80% B. The electrospray ionization (ESI) source was combined with a mass spectrometer (ESI-QTOF-MS) to complete the mass spectrometry detection, and both positive and negative ions served as the ion scanning mode. The capillary voltage was 2.0 Kv. Nitrogen was used as the drying gas and atomizing gas, the temperature and flow rate of the drying gas were 500°C and 13 L/min, respectively, the scanning speed was 0.2 s, and the leucine enkephalin solution was used as an external standard to calibrate the relative molecular mass. Argon was used as the collision gas with a flow rate of 0.8 L/min, and the CID was cracked. The mass scanning range was 50–2000 Da. Data collection and processing were carried out with the Waters UNIFI Scientific Information System. The external standard method was used to quantify the quality control components (pedunculoside, syringin) of IR. Linear regression for the calibration curve and the value of the coefficient of determination were y = 40130x + 5221, y = 10600x + 6805; and R^2^ = 0.9948, R^2^ = 0.9902. Finally, the IR (0.4 mg) was dissolved in 50% methanol (1 ml), sonicated for 30 min, and passed through a 0.22 µm filter for quantification.

### Animal Treatment

Sixty specific pathogen-free C57BL/6 J male mice, weighing 21–24 g, were obtained from the Air Force Medical University Experimental Animal Center (Xi’an, China). All animal experimental procedures were approved by the Laboratory Animal Welfare and Ethics Committee of Air Force Medical University (No. 20191206). Mice were randomly divided into six groups with 10 mice, including control, DSS, IR-0.45 g/kg, IR-0.9 g/kg, and IR-1.8 g/kg, and positive control (balsalazide; Hubei Baikehendi Pharmaceutical Company, Hubei, China) groups. The control group drank tap water and the other five groups drank 2.0% DSS [molecular weight (MW): 36,000–50,000; MP Biomedicals, LLC, Irvine, CA, United States] for 7 days followed by 2 weeks of tap water as one cycle, which was repeated for three cycles. The last cycle of drinking tap water for 2 weeks was adjusted to 1 week (Wirtz et al., 2017). At the beginning of the second modeling cycle, mice in the IR-0.45 g/kg, IR-0.9 g/kg, and IR-1.8 g/kg groups were gavaged with IR at 0.45, 0.9, and 1.8 g/kg, respectively, and the positive control group was given balsalazide at 3.4 g/kg. The dosage of balsalazide was calculated based on the human clinical dosage (mouse dosage = human daily dosage/60 kg body weight × 9.1) (Wei, 2002). During the treatment period, the body weight of mice was measured and recorded daily. The specific experimental steps are shown in Figure 2A.

### Disease Activity Index Score

The behavioral state and stool morphology of the mice were observed, and the disease activity index (DAI) score was evaluated by comprehensively scoring the degree of weight loss, stool characteristics and morphology, and state of hematochezia. The detailed scoring criteria are shown in Table 1 (Zhou et al., 2020).

**TABLE 1:** DAI scoring standards of UC mice.

Weight loss (%)	Stool morphology	Hematochezia	Score
0	Normal	Normal	0
1–5	Loose stools	Positive occult blood	1
5–10	Loose stools	Visible mild bloody stools	2
10–15	Mucous stools	Visible bloody stools	3

### Histopathology Analysis

Hematoxylin and eosin staining (H & E) was used to evaluate the histopathological changes of the colon. The colon tissue was fixed in paraformaldehyde, dehydrated, and embedded in a paraffin block. After the sections were cut into 3–5 µm slices, H & E staining was performed and sections were observed under a microscope at ×40 and ×200 magnifications. Histopathological scoring was previously reported for the degree of inflammatory cell infiltration, crypt destruction, and scope of the lesion (Zhang et al., 2018).

### Blood Routine Examination

Blood (50 µl) was collected from the venous plexus of the fundus and placed in a 0.5 ml tube with EDTA anticoagulation. Then it was analyzed with a fully automatic hematology analyzer to detect white blood cells (WBCs), red blood cells (RBCs), and hemoglobin (HGB) in the whole blood.

### Quantitative Polymerase Chain Reaction Analysis

Total RNA from colon tissue was extracted using a Total RNA Kit (Omega Bio-Tek, Inc., Guangzhou, China). cDNA was synthesized by QuantiNova SYBR Green PCR Kit (Qiagen, Germany). Then the cDNA was used to determine the mRNA levels of IL-1β, TNF-α, and IL-6 through quantitative polymerase chain reaction (qPCR). β-actin was used as an internal control. The primer sequences used for qPCR were designed by Shanghai Sangon Biotech Co., Ltd. (Shanghai, China) and are shown in Supplementary Table S1.

### Detection of Serum Lipopolysaccharide

An enzyme-linked immunosorbent assay (ELISA) kit for LPS (Cloud-Clone Co., Houston, TX, United States) was employed to detect the concentration of LPS in the serum of mice. According to the instructions, six concentration standards were diluted and 50 µl of each dilution of standard, blank, and samples was added to the pre-coated 96-well plate. Then 50 µl Detection Reagent A was immediately added to each well and incubated for 1 h at 37°C. Each well was washed with 350 µl of ×1 Wash Solution, followed by the addition of 100 µl Detection Reagent B and incubation for 30 min at 37°C. The wash process was repeated and then 90 µl Substrate Solution was added to each well and incubated for 10–20 min at 37°C, followed by the addition of 50 µl Stop Solution and measurement by a microplate reader at 450 nm.

### Detection of Serum Myeloperoxidase

Myeloperoxidase (MPO) content was closely related to the severity of UC. The Mouse MPO ELISA Kit was used to detect the content of MPO in the serum of mice according to the manufacturer’s instructions (MultiSciences (Lianke) Biotech Co., Ltd., China).

### Detection of Serum Fluorescein Isothiocyanate

At the end of the experiment, mice were gavaged with 0.6 mg/g fluorescein isothiocyanate (FITC)-dextran (MW: 40 kDa; TdB Labs AB, Uppsala, Sweden), and blood was collected from the venous plexus of the fundus of the mouse after 4 h. The serum FITC content was measured by a fluorescence microplate reader.

### Alcian Blue Staining

The paraffin slides of the colon tissue were dewaxed and stained with Alcian blue dye solution A for 10–15 min. After washing with tap water, the slides were stained with Alcian blue dye solution B for 3 min. The slides were observed under ×100 magnification.

### Terminal Deoxynucleotidyl Transferase dUTP Nick-End Labeling Analysis

Proteinase K working solution was added to the paraffin slides and incubated at 37°C for 25 min, followed by permeabilization and equilibrium at room temperature. Take appropriate amount of terminal deoxynucleotidyl transferase enzyme, dUTP, and buffer in the terminal deoxynucleotidyl transferase dUTP nick-end labeling (TUNEL) kit were mixed at a 1:5:50 ratio and incubated on the tissue at 37°C for 2 h. The nucleus was stained with DAPI. The excitation was measured at 465–495 nm and the emission was measured at 515–555 nm with a fluorescence microscope (Nikon).

### Detection of Oxidative Stress Indexes

Colon tissues was fully homogenized with extraction reagent on ice, centrifuged at 8000 g for 10 min to separate the supernatant, and the protein concentration was determined by BCA quantitative kit. Then the glutathione (GSH), oxidized glutathione (GSSG), and malondialdehyde (MDA) levels were measured by micro GSH assay kit, micro GSSG assay kit, and MDA assay kit according to the instructions, respectively (Solarbio Science and Technology, Beijing, China). Then the GSH/GSSG ratio was calculated.

### Immunohistochemical Analysis

After antigen retrieval, paraffin sections were blocked in bovine serum albumin for 30 min and incubated overnight at 4°C with primary antibodies against E-cadherin and Ki-67 (Servicebio, Wuhan, China). After washing slides three times with phosphate-buffered saline, slides were incubated with horseradish peroxidase-labeled secondary antibody in the dark for 50 min followed by the addition of DAB for color development and microscopy (Nikon).

### Cell Culture

The Caco2 cell line was obtained from Xijing Hospital of Digestive Diseases (Shaanxi, China), cultured in high-glucose Dulbecco’s modified Eagle medium (DMEM) (Meilunbio, Beijing, China) medium supplemented with 10% fetal bovine serum and 1% penicillin-streptomycin antibiotics, and maintained at 37°C in 5% CO_2_.

### Cell Viability Assay

Cell Counting Kit-8 (CCK-8) (Glpbio, Montclair, CA, United States) was used to detect cell viability. Briefly, 100 µl Caco2 cell suspension (5 × 10^4^ cells/ml) was added to each well of a 96-well plate and cultured overnight. Then DMEM was replaced with a medium containing different concentrations of IR or LPS and incubated for 12, 24, and 48 h. Then 10 µl CCK-8 per 100 µl DMEM was added to the wells and incubated at 37°C for 2 h. The absorbance was detected at 450 nm.

### Establishment of Colitis Cell Model

Caco2 cells (1 × 10^6^ cells/well) were seeded in 6-well plates and cultured overnight. The culture medium was replaced with a medium containing 1 μg/ml LPS (Sigma-Aldrich, St. Louis, MO, United States). After incubating for 4, 8, 12, and 24 h, total cell protein was extracted, and the protein expression of oncostatin M (OSM), OSM receptor (OSMR), and IL-6 was detected by western blot analysis to determine the best modeling time.

### Western Blot Analysis

Proteins from the colon tissues of mice or Caco2 cells were separated by sodium dodecyl sulfate-polyacrylamide gel electrophoresis and electro transferred to PVDF membranes. Then membranes were blocked in 5% non-fat milk and incubated overnight at 4°C with the following primary antibodies: anti-signal transducer and activator of transcription 3 (STAT3) (WL01836; Wanleibio, Shenyang, China), anti-phosphorylated STAT3 (p-STAT3) (#9145; Cell Signaling Technology, Danvers, MA, United States), anti-IL-6 (WL02841; Wanleibio), anti-Keap-1 (WL03285; Wanleibio), anti-Nrf2 (WL02135; Wanleibio), anti-heme oxygenase 1 (HO-1) (WL02400; Wanleibio), anti-zonula occludens-1 (ZO-1) (WL03419; Wanleibio), anti-occludin (WL01996; Wanleibio), anti-claudin-1 (WL03073; Wanleibio), anti-OSM (PA5-81453; Invitrogen, Carlsbad, CA, United States), and anti-OSMR (ab210771; abcam, Cambridge, MA, United States); anti-β-actin (AT0001; Engibody Biotechnology, Inc., Milwaukee, United States) served as the internal control. Then membranes were incubated with the appropriate secondary antibody for 1 h at room temperature. The intensity of each band was scanned by the ChemiDoc™XRS+ Imaging System and analyzed with ImageJ software.

### Statistical Analyses

Data were analyzed using SPSS 23.0 software (IBM SPSS Statistics, Armonk, NY, United States). One-way analysis of variance was performed with equal variances; otherwise, nonparametric tests were performed. The data are presented as the mean ± standard deviation, with *p* < 0.05 considered significantly different.

## Results

### Chemical Analyses of *Ilex rotunda Thunb*

**FIGURE 1:**
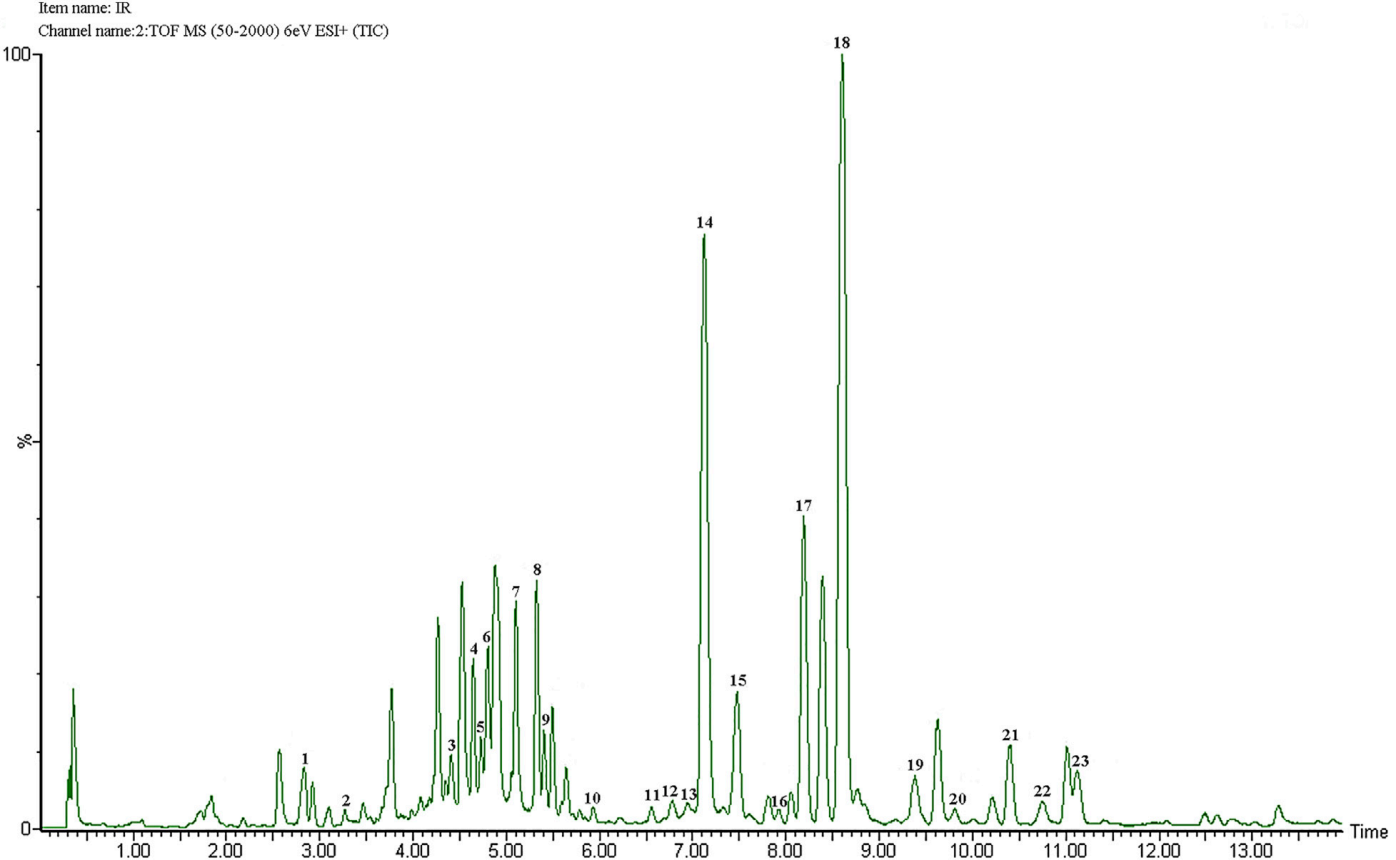
TIC of IR in positive ion mode.

The components of IR were analyzed by ultra-performance liquid chromatography-quadrupole time-of-flight mass spectrometry.

(UPLC-QTOF-MS.). The total ions chromatogram (TIC) in positive ion mode is shown in Figure 1. Combining the literature and mass spectrometry MS information including molecular weight and fragment ion peaks, we have confirmed a total of 23 components in IR, and the results were shown in (Table 2.). In addition, pedunculoside and syringin as the quality control components of IR were quantified by external standard quantitative analysis, and the contents of pedunculoside and syringin were 18.8 μg/mg and 6.5 μg/mg, respectively.

**TABLE 2:** Main components of IR identified by UPLC-QTOF-MS.

No	t_R_ (min)	Measured (m/z)	Error (ppm)	Formula	Fragment ions (m/z)	Identification
1	2.83	372	1.5	C_17_H_24_O_9_	353.1294, 193.1090, 163.0585, 161.0795	Syringin
2	3.27	370	3.6	C_17_H_22_O_9_	209.1069, 177.0772	Sinapaldehyde glucoside
3	4.41	426	2.9	C_20_H_26_O_10_	325.1310, 163.0585	Caffeic acid (1-hydroxyl-4-O-β-D-glucopyranosylprenyl)-ester
4	4.65	580	1.2	C_28_H_36_O_13_	573.2634, 402.2126, 401.2088, 383.1967, 330.1488	(-)-Syringaresinol-4-O-beta-D-glucopyranoside
5	4.72	640	3.2	C_29_H_36_O_16_	581.2554, 559.2475, 479.2119, 443.1870, 277.1040, 263.1234, 163.0601	Kelampayoside B
6	4.81	426	5.1	C_20_H_26_O_10_	247.1272	4-caffeoyl-3-methyl-but-2-ene-1,4-diol 1-O-β-D-glucopyranoside
7	5.11	604	−2.6	C_35_H_56_O_8_	340.2998, 279.2770	3β-[(α-L-arabinopyanosyl) oxy]-19α-hydroxyolean-12-en-28-oic acid
8	5.33	604	−1.3	C_35_H_56_O_8_	425.1754, 397.3508, 396.8493, 263.1234, 163.0585	Ziyuglycoside II
9	5.41	678	−4.1	C_37_H_58_O_11_	500.1883, 499.1853, 445.2390	19α,23-dihydroxyurs-12-en-28-oic acid
10	5.94	812	5.1	C_42_H_68_O_15_	703.4561, 673.4752, 645.0314, 623.0475, 487.3038, 429.2686, 241.1711	Ilexoside XLI
11	6.57	810	2.5	C_42_H_66_O_15_	805.5347, 671.4646, 455.4050, 437.3941, 435.3761	Ilexoside XLVIII
12	6.78	664	4.8	C_36_H_56_O_11_	503.3972, 485.3865, 439.3741, 279.1262	Ilexasprellanoside D
13	6.90	780	3.2	C_42_H_68_O_13_	671.4613, 641.4467, 451.3751, 398.2888, 376.3059, 282.2395	3-O-β-D-glucopyranosyl-oleanolic acid 28-O-β-D-glucopyranoside
14	7.12	650	0.6	C_36_H_58_O_10_	471.4030, 453.3918, 435.3788, 407.3809	pedunculoside
15	7.48	928	2.0	C_47_H_76_O_18_	789.5362, 657.4775, 560.3391, 487.2982, 455.4077, 437.3941	Ilexoside K
16	7.92	766	3.9	C_41_H_66_O_13_	657.4775, 456.4119, 455.4077, 437.3941	Ziyuglycoside I
17	8.19	650	1.5	C_36_H_58_O_10_	471.4030, 453.3918, 435.3788	Ilexoside V
18	8.60	664	0.9	C_36_H_56_O_11_	503.3972, 485.3865, 467.3714, 457.3873, 439.3741, 421.36	Ilexsaponin A1
19	9.38	488	2.3	C_30_H_48_O_5_	471.4057, 454.3965, 453.3918, 436.3818, 435.3788,288.3236	Rutundic acid
20	9.82	810	1.8	C_42_H_66_O_15_	671.4613, 655.4648, 455.4104, 437.3941, 327.1188, 299.1984, 277.2140	Ilekudinoside B
21	10.40	794	1.8	C_42_H_66_O_14_	655.4615, 641.4821, 440.4138, 439.4114, 431.2565, 191.2025	Scheffleside L
22	10.75	488	2.5	C_30_H_48_O_5_	471.4057, 453.3918, 436.3818, 435.3788, 407.3809	Rotungenic acid
23	11.12	502	1.7	C_30_H_46_O_6_	485.3865, 467.3741, 439.3741, 421.3627	Ilexgenin A

### *Ilex rotunda Thunb* Significantly Improves the Symptoms of Dextran Sulfate Sodium-Induced Ulcerative Colitis

**FIGURE 2:**
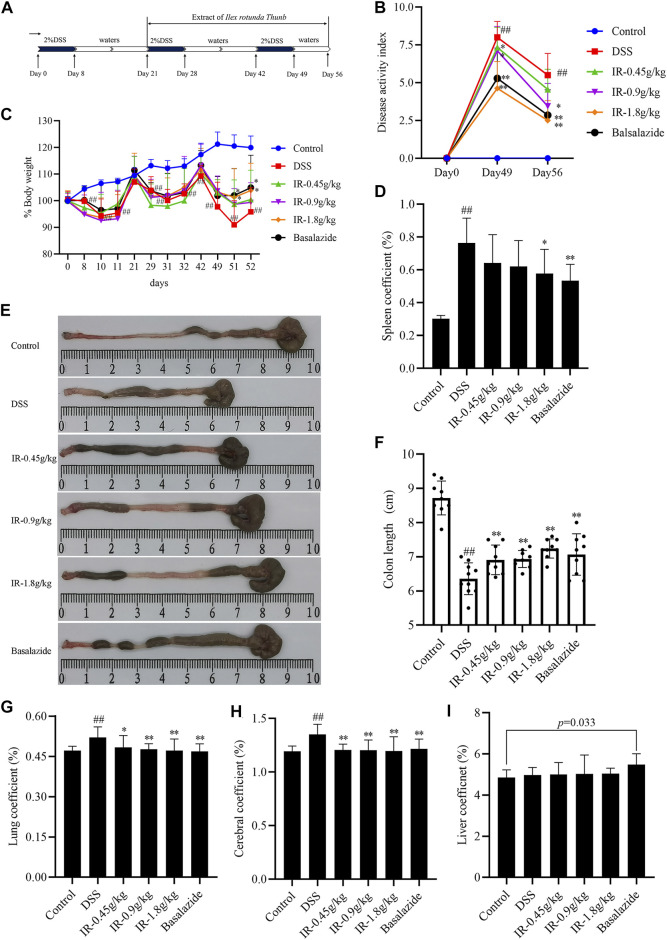
IR alleviates the pathological symptoms of DSS-induced chronic UC mice. **(A)** Experimental design. **(B)** Disease activity index of each group. **(C)** The body weight change of mice in each group during the experiment. **(D)** Spleen coefficient of mice in each group. **(E)** Representative colon images at day 56. **(F)** Statistical graph of colon length. **(G)** Lung coefficient. **(H)** Cerebral coefficient. **(I)** Liver coefficient. All data were compared using one-way ANOVA, and *p*-values reflected differences between experimental groups (*n* = 9).

After drinking 2% DSS combined with tap water for three cycles, compared with the control group, the body weight of the mice in the DSS group was reduced, and the difference was statistically significant since day 29 of modeling (*p* < 0.01; Figure 2C). The body weight of mice in the IR-1.8 g/kg group and balsalazide group was higher than that of the DSS group on days 51 and 52 (*p* < 0.05), but IR-0.45 g/kg and IR-0.9 g/kg did not improve the body weight of the UC mice (*p* > 0.05; Figure 2C). The DAI score is an important indicator for evaluating the success of the UC model. Compared with the control group, the DAI score of mice in the DSS group was increased (*p* < 0.01). On day 49, the mucopurulent bloody stool was obvious (Figure 2B). By day 56, although the weight had rebounded, the bloody stool still existed, indicating that the chronic UC model was successfully established. Compared with the DSS group, IR improved the bloody stool of UC mice and reduced the DAI score, with statistical significance between the IR-0.9 and IR-1.8 g/kg groups (*p* < 0.05 and *p* < 0.01, respectively; Figure 2B). We found that the colon length was shortened, and the spleen, lung, and brain coefficients were increased in the DSS group compared to the control group on day 56 (Figures 2D–H). Administration of IR reversed all of these effects in a dose-dependent manner (Figures 2D–H). In the balsalazide group, the liver coefficient of mice was higher than that of the control group when it exerted therapeutic effects on UC mice, which suggests that it may cause liver damage during long-term use (Figure 2I). We also observed the preventive and therapeutic effects of IR on acute UC mice and found that IR improved the weight loss and colon shortening of acute UC mice in a dose-dependent manner; and reduced the DAI score and spleen, lung, and brain coefficients (Supplementary Figure S1).

### *Ilex rotunda Thunb* Alleviates the Inflammation of Dextran Sulfate Sodium-Induced Ulcerative Colitis Mice

**FIGURE 3:**
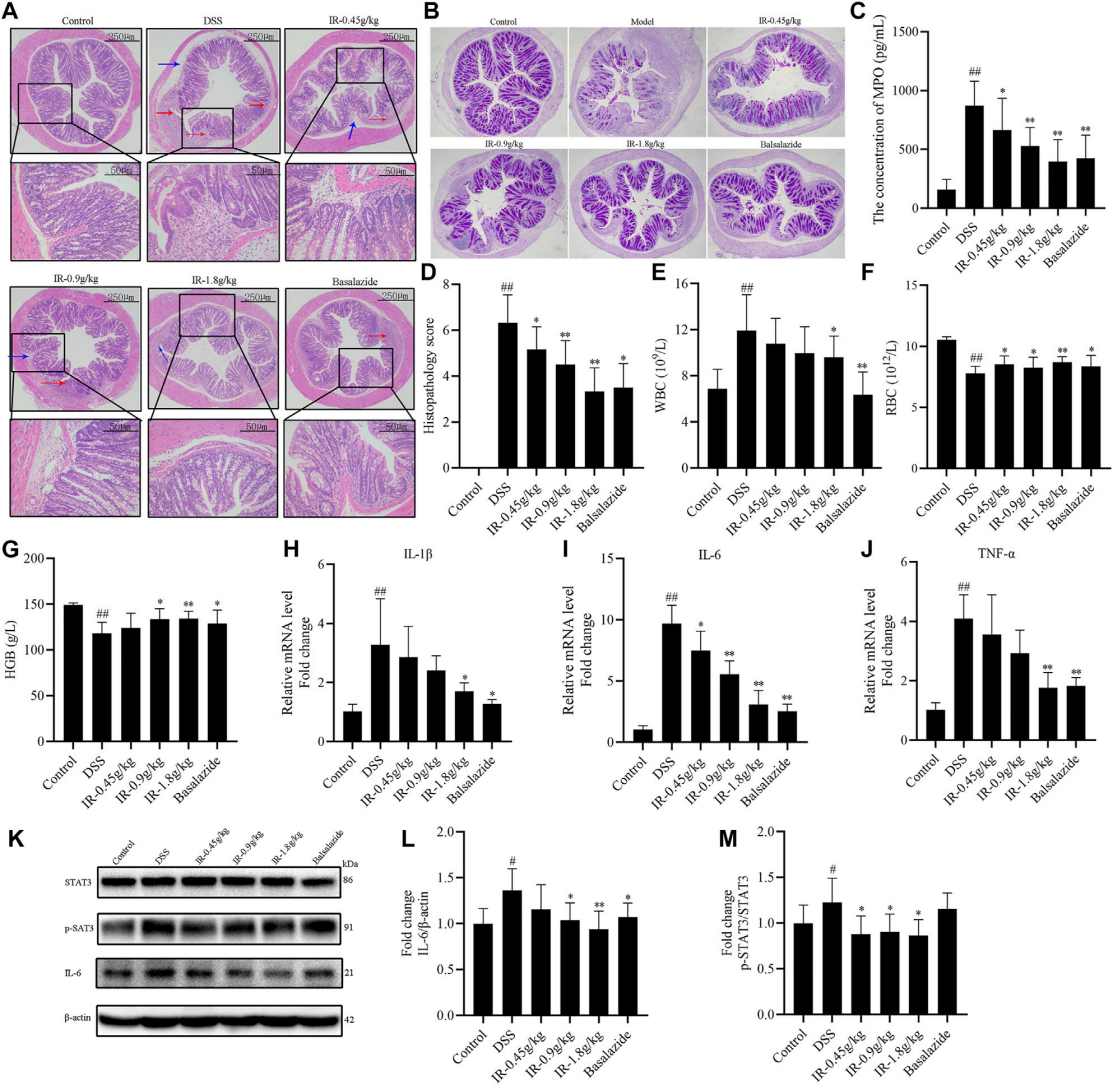
IR suppress inflammation of UC mice. **(A)** Representative H & E staining colon tissue of mice (day 56). **(B)** PAS staining colon tissue of mice (day 56). **(C)** MPO content was measured by ELISA in serum of mice. **(D)** Histopathology scores. **(E)** The number of WBC in each group of mice tested by routine analysis of blood. **(F)** The number of RBC in each group of mice. **(G)** The concentration of HGB in each group of mice tested by routine analysis of blood. **(H–J)** The mRNA levels of IL-1β, IL-6, and TNF-α from the colon of mice. **(K)** The protein levels of STAT3, p-STAT3, and IL-6 in colon detected by western blotting. **(L)** The gray intensity analysis of IL-6. **(M)** The gray intensity analysis of p-STAT3/STAT3. All data were compared using one-way ANOVA, and *p*-values reflected differences between experimental groups.

Histopathological examination showed that the colon cells of the control group were tightly arranged, with no pathological changes. In the DSS group, we observed that the tissue structure was disordered, the inflammatory cells infiltrated into the muscle layer, the goblet cell numbers were reduced, and the crypts disappeared accompanied by submucosa edema (Figures 3A,B,D). Compared with the DSS group, the IR-0.45 g/kg group had partial recovery of the colon structure and increased goblet cells and submucosal edema, but it had more areas of inflammatory cell infiltration. The colons of mice in the IR-0.9 g/kg, IR-1.8 g/kg, and balsalazide groups were infiltrated with few inflammatory cells, and the tissue structure was restored (Figures 3A,B,D, Supplementary Figure S2A). MPO is positively correlated with the inflammatory response of UC. Detection of the MPO in the serum showed that compared with the control group, the MPO content of mice in the DSS group was increased (*p* < 0.01; Figure 3C). After treatment with different dosages of IR, the MPO in the serum of UC mice was reduced in a dose-dependent manner (*p* < 0.05 or *p* < 0.01; Figure 3C). Routine blood analysis found that the number of WBCs in the whole blood of mice of the DSS group was higher than that of the control group, indicating that the mice had an obvious inflammatory response (Figure 3E, Supplementary Figure S2B). Administering IR and balsalazide reduced the WBCs of chronic and acute UC mice, and the IR-1.8 g/kg and balsalazide groups were statistically significant compared with the DSS group for the treatment of chronic UC mice (Figure 3E, Supplementary Figure S2B). In addition, routine blood tests also found that the RBCs and HGB of mice in the DSS group were lower than those in the control group, and IR also increased the RBCs and HGB in the whole blood of chronic and acute UC mice compared with the DSS group (Figures 3F,G, Supplementary Figures S2C,D).

To further identify the inflammatory response of mice, the mRNA levels of inflammatory cytokines including IL-1β, IL-6, and TNF-α were measured and found to be increased in the DSS group compared with the control group; whereas IL-1β, IL-6, and TNF-α levels were decreased after treatment with IR (*p* < 0.05 or *p* < 0.01) (Figures 3H–J, Supplementary Figures S2E–H). The detection of inflammatory-related proteins in the colon tissue showed that compared with the control group, the expression of p-STAT3/STAT3 and IL-6 proteins in the DSS group was increased, and IR treatment reduced the expression of p-STAT3/STAT3 and IL-6 proteins in the colon tissue of chronic and acute UC mice (*p* < 0.05 and *p* < 0.01, respectively) (Figures 3K–M, Supplementary Figures S2I–K). Otherwise, balsalazide could reduce the expression of IL-6 protein in the colon, but did not affect the expression of p-STAT3/STAT3 proteins (Figures 3K–M, Supplementary Figures S2I,K).

### *Ilex rotunda Thunb* Attenuates the Oxidative Stress and Apoptosis of Colon Tissue in Dextran Sulfate Sodium-Induced Mice

**FIGURE 4:**
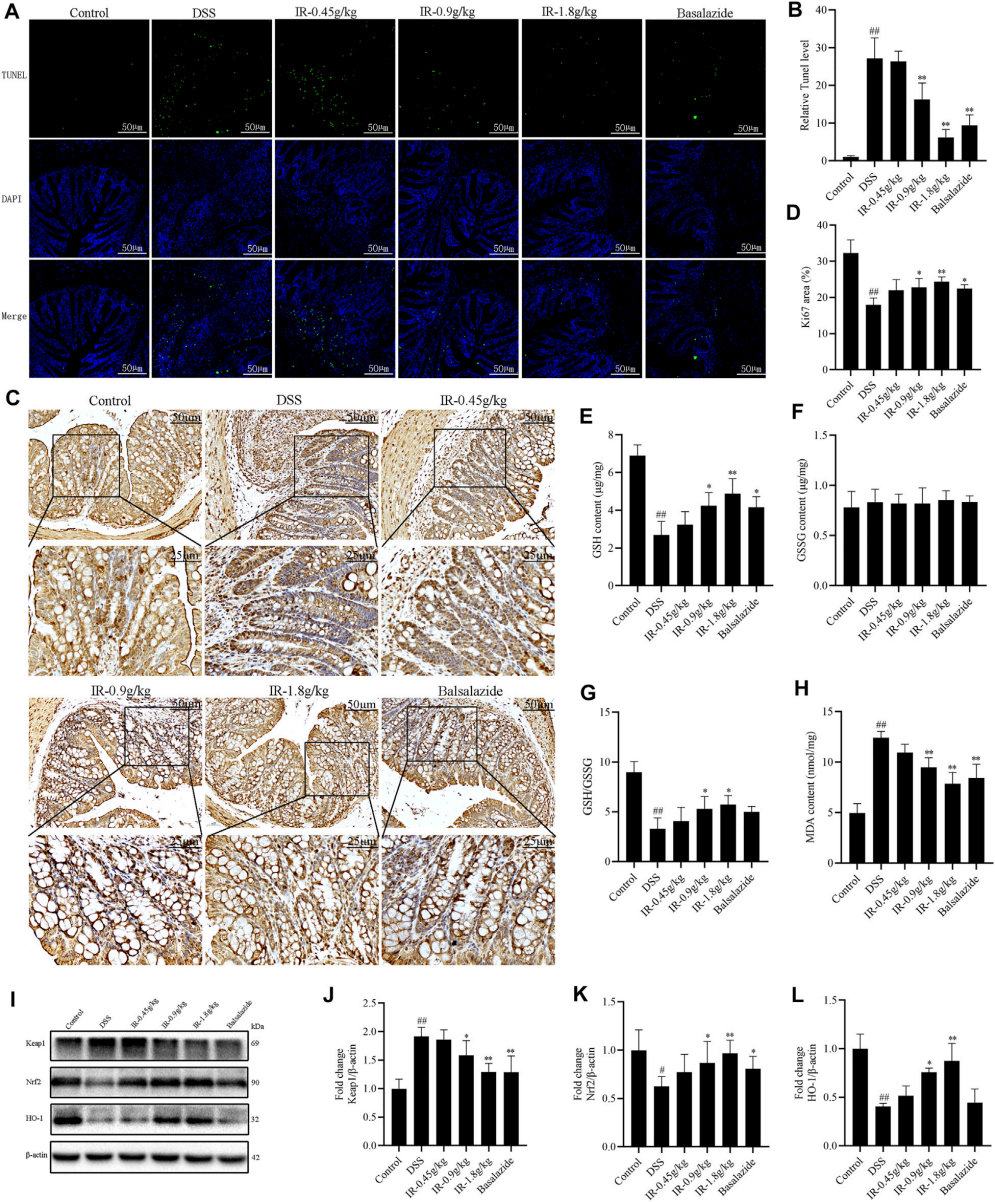
IR alleviates the apoptosis and oxidative stress of colon tissue in UC mice. **(A)** Colon cell apoptosis detected by Tunel staining. **(B)** Positive area statistics of Tunel. **(C)** Colonic epithelial cell proliferation detected by Ki67 staining. **(D)** Histochemical positive area statistics of Ki67. **(E,F)** The content of glutathione (GSH) and oxidized glutathione (GSSG). **(G)** The ratio of GSH/GSSG. **(H)** The content of malondialdehyde (MDA). **(I)** The protein levels of Keap1, Nrf2, and HO-1 in colon detected by western blotting. **(J-L)** The gray intensity analysis of Keap1, Nrf2, and HO-1 proteins, respectively. All data were compared using one-way ANOVA, and *p*-values reflected differences between experimental groups (*n* = 3).

TUNEL and Ki67 staining were employed to evaluate the apoptosis and proliferation of colon tissues. The results showed that compared with the control group, there was a large number of apoptotic cells and fewer proliferating cells in the intestinal epithelium of the DSS group, which was consistent with the destruction of the tissue structure and the appearance of a large number of necrotic areas observed in (Figures 3, 4A–D–D). Compared with the DSS group, mucosal layer proliferating cells were less in IR-0.45 g/kg-treated mice, but plenty of apoptotic cells still existed, while IR-0.9 g/kg and IR-1.8 g/kg and balsalazide treatment reduced colon apoptotic cells, and promoted the proliferation of mucosal cells (Figures 4A–D). The imbalance between oxidation and antioxidation triggers oxidative stress, and excessive oxidative stress can cause inflammatory infiltration of neutrophils and cell necrosis. The intracellular GSH, GSSG, and MDA contents could well reflect the redox state and lipid oxidation level of the organism. The results showed that compared with the control group, the GSH content and GSH/GSSG ratio of colon in mice of DSS group decreased and the MDA content increased significantly, while the GSH content and GSH/GSSG ratio were increased in the colon of mice in IR-0.9 g/kg and IR-1.8 g/kg groups, and the MDA contents decreased, indicating that IR treatment could improve the antioxidant capacity of UC mice and reduce their lipid oxidation levels (Figures 4E–H). Furthermore, detecting the expression of oxidative stress-related proteins such as Keap-1, Nrf2, and HO-1 showed that, compared with the control group, the expression of Keap-1 protein increased, accompanied by the decreased expression of Nrf2 and HO-1 proteins in mice of the DSS group, indicating that the colon of UC mice had excessive oxidative stress. IR treatment alleviated the oxidative stress of UC mice by reducing Keap-1 protein expression and increasing Nrf2 and HO-1 protein expression (Figures 4I–L).

### *Ilex rotunda Thunb* Protects Against Injury to the Intestinal Mucosal Barrier

**FIGURE 5:**
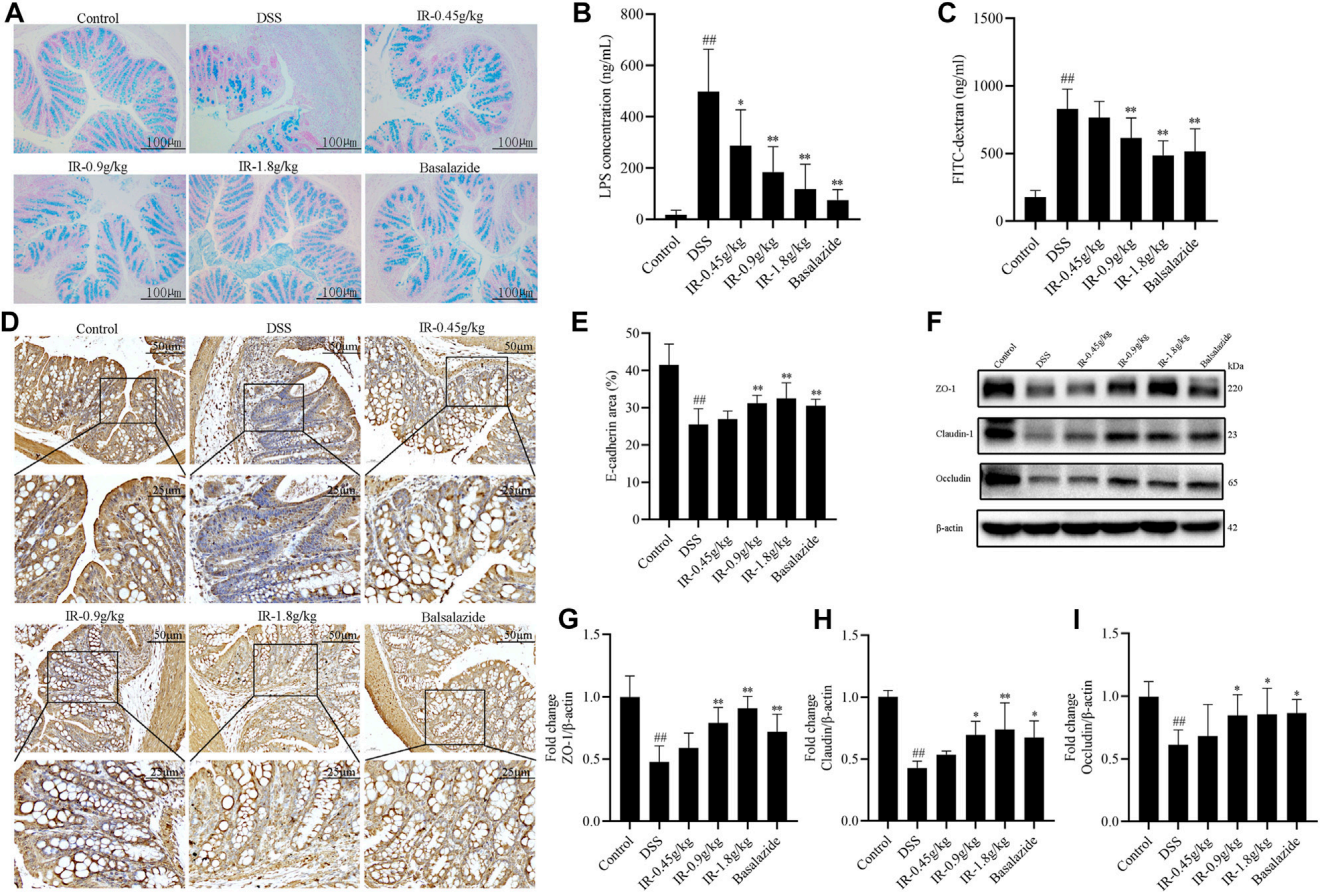
IR alleviates intestinal mucosal barrier damage in UC mice. **(A)** Detection of acidic mucus layer in colon of mice by alcian blue staining (*n* = 6). **(B)** The LPS content in serum of mice (*n* = 6). **(C)** The FITC-dextran content in serum of mice (*n* = 6). **(D)** Immunohistochemical detection of E-cadherin protein expression in colon of mice (*n* = 3). **(E)** Histochemical positive area statistics of E-cadherin (*n* = 3). **(F)** The protein levels of ZO-1, claudin-1, and occludin in colon detected by western blotting. **(G–I)** The gray intensity analysis of ZO-1, claudin-1, and occludin proteins, respectively (*n* = 3). All data were compared using one-way ANOVA, and *p*-values reflected differences between experimental groups.

The intestinal mucus layer and intercellular tight junction are important components of the intestinal mucosal barrier. Alcian blue staining was used to detect the integrity of the colon mucus layer due to its ability to combine with acidic groups to make the acidic mucous on the colon appear blue. The results showed that the colon mucus layer of the control group was intact and distributed neatly on the cell surface, and the colon mucus layer of the DSS group was destroyed and disappeared in some areas of the colon tissue (Figure 5A). After IR treatment, the colonic mucous layer of chronic and acute UC mice was restored in a dose-dependent manner (Figure 5A, Supplementary Figure S3A). After the intestinal mucosal barrier was damaged, the mucosal permeability was increased, and LPS produced by intestinal flora and FITC given by gavage were more likely leaks into the blood from the intestine. Compared with the control group, the LPS and FITC in the serum of mice in the DSS group were significantly increased, and IR reduced the LPS in the serum of acute and chronic UC mice (*p* < 0.05 or *p* < 0.01) (Figure 5B, Supplementary Figure S3B). Furthermore, IR reduced FITC from the intestine into the serum of chronic UC mice (Figure 5C). Adhesion junction proteins such as E-cadherin and tight junction proteins including occludin, claudin-1, and ZO-1 are the main parts of cell-cell junctions. Compared with the control group, the expression of E-cadherin, occludin, claudin-1, and ZO-1 proteins in the colon of the DSS group was significantly reduced, and the intestinal mucosal barrier was damaged (Figures 5D–I). IR partially restored the reduced expression of E-cadherin, occludin, claudin-1, and ZO-1 proteins to protect the integrity of the intestinal mucosal barrier (Figures 5D–I, Supplementary Figures S3C–F).

### *Ilex rotunda Thunb* Alleviates Colitis by Modulating the Oncostatin M/Oncostatin M Receptor Pathway

**FIGURE 6:**
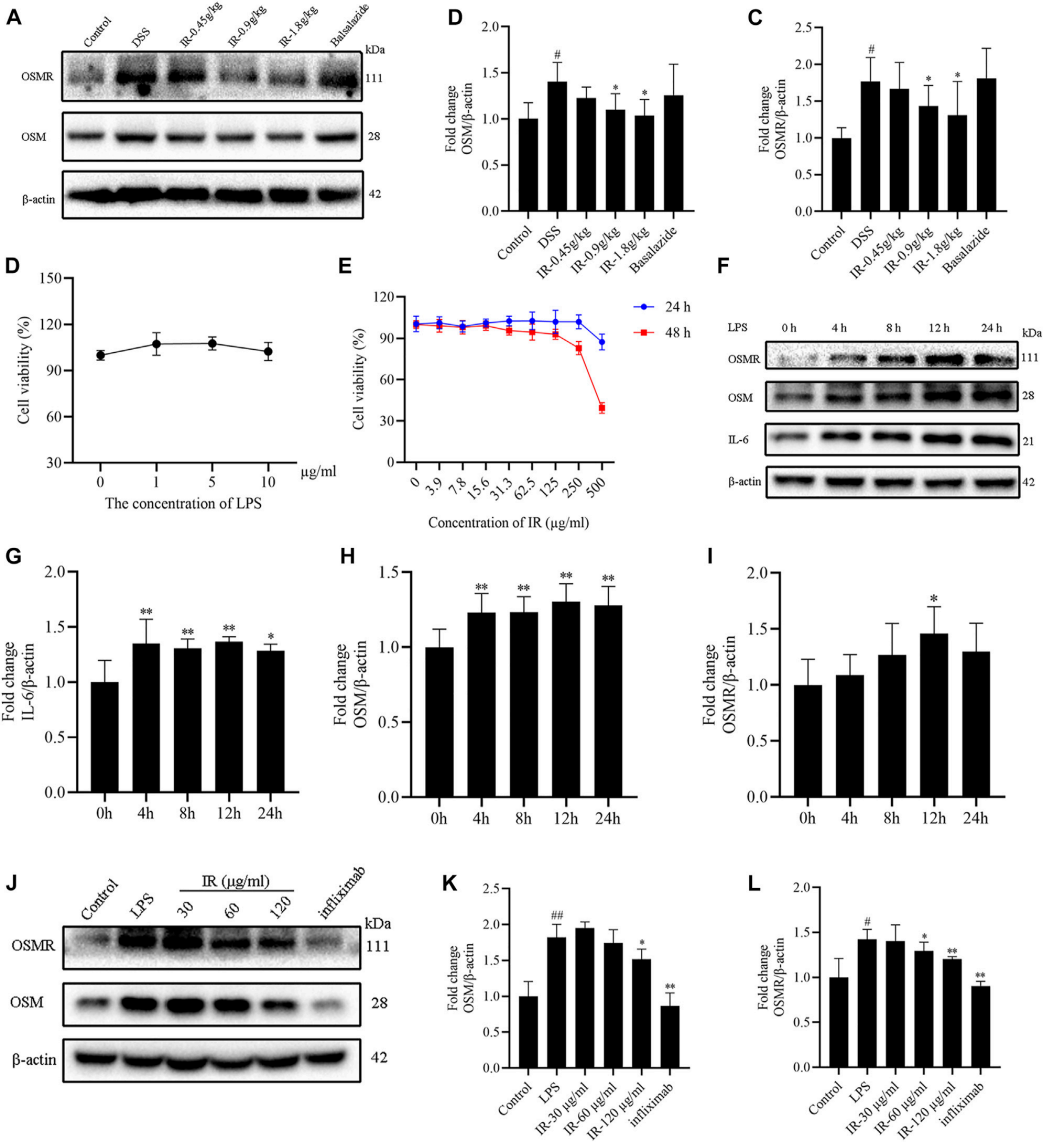
IR ameliorates UC in mice by regulating OSM/OSMR pathway. **(A)** The protein levels of OSM and OSMR in colon of mice detected by western blotting, with gray intensity analysis shown in panel **(B,C)**. **(D)** Cell viability of Caco2 cells treated with different concentrations of LPS for 24 h. **(E)** Cell viability of Caco2 cells treated with different concentrations of IR for 24 and 48 h. **(F)** The expression of OSM, OSMR, and IL-6 proteins of Caco2 cells detected by western blotting after 1 μg/ml LPS stimulated, with gray intensity analysis shown in panel **(G–I)**. **(J)** The effect of IR on the expression of OSM and OSM proteins of Caco2 cells after LPS stimulated, with gray intensity analysis shown in panel **(K,L)**. All data were compared using one-way ANOVA, and *p*-values reflected differences between experimental groups (*n* = 3).

OSM is an important member of the IL-6-related cytokine subfamily and is closely related to the occurrence and development of UC (Rose-John, 2018). The results indicated that compared with the control group, the protein expression of OSM and OSMR was increased in the colon of mice of the DSS group (*p* < 0.05), consistent with our previous study on acute UC mice (Li Y. et al., 2021). Compared with the DSS group, IR treatment reduced the expression of OSM and OSMR proteins (Figures 6A–C).

LPS was used to stimulate Caco2 cells to establish a colitis model. We found that 1, 5, 10 μg/ml LPS did not affect the viability of Caco2 cells, whereas 1 μg/ml LPS stimulated Caco2 cells for 0, 4, 8, 12, and 24 h. The expression of IL-6, OSM, and OSMR proteins in the cells increased along with prolonged action time, peaking at 12 h. Therefore, we chose 1 μg/ml LPS to stimulate Caco2 cells for 12 h to establish a cell colitis model (Figures 6F–I). The cytotoxicity of IR (0–500 μg/ml) was tested in Caco2 cells for 24 and 48 h, and the results showed that an IR concentration below 250 μg/ml did not affect the viability of Caco2 cells. Hence, 30, 60, and 120 μg/ml IR were selected to pretreat Caco2 cells for 12 h. Then the cells were cultured for an additional 12 h in a fresh medium containing 1 μg/ml LPS, followed by the detection of OSM and OSMR proteins in cells. The different dosages of IR decreased the expression of OSM and OSMR proteins caused by LPS stimulation (Figures 6J–L). These results indicate that IR can alleviate UC by regulating the OSM/OSMR pathway.

## Discussion

The pathogenesis of UC is complex, involving the interaction of genetic susceptibility, environmental factors, gut microbial disorders, and disturbances in immune homeostasis. With economic development and lifestyle changes in developing countries, the incidence of UC has been increasing in countries in Africa, Asia and South America (Ng et al., 2017). Because of the long and indolent course of UC, the frequent alternation between active and remission phases of the disease, and the high rate of colorectal cancer transformation, the work and life of UC patients are seriously affected (Gajendran et al., 2019; Damas and Abreu, 2020). Despite the great progress has been made in the understanding and treatment of UC, some patients still lose response or intolerance to UC therapeutic drugs in clinical application, about 15% of patients still need to undergo colectomy, and 5-ASA therapeutic drugs such as mesalazine and biological agents such as infliximab are accompanied by adverse effects in the process of use (Magro et al., 2012; Sehgal, et al., 2018). Thus, it is important to develop new drugs that can effectively treat UC from natural products.

In the Jiangxi “Handbook of Herbal Medicine,” there is a record that IR can be used to treat gastric and duodenal ulcers. Previous studies have pointed out that Kuijieling, which uses IR as the “Jun drug,” can exert therapeutic effects on UC by regulating the differentiation of regulatory T cells and T helper 17 cells and improving the intestinal flora of mice (Long et al., 2018; Pan et al., 2020). Our previous research also demonstrated that FYC, with IR as one of the important prescription drugs, can alleviate UC by regulating OSM/OSMR and improving intestinal flora (Li X. X. et al., 2021). However, it is still unclear whether IR has a therapeutic effect on UC and the underlying mechanism of action is unknown.

In this study, we analyzed the components of IR with UPLC-QTOF-MS, identified 23 main components through literature and standard substance comparison, and determined the contents of syringin and pedunculoside. Most UC patients have recurrent attacks, with alternating periods of active and remission, requiring long-term drug treatment and maintenance in clinical practice. DSS can induce a UC model that is similar to human UC in both immunological and pathological manifestations. The chronic UC model induced by DSS is established by treatment with DSS for 7 days and tap water for 14 days as a cycle, repeated for three cycles. It can better simulate the alternating occurrence of the active phase and remission phase in the clinic (McQueen et al., 2019). However, as the time of drinking tap water was prolonged, symptoms such as bloody stool and weight loss of the mice gradually decreased. To be more conducive to the observation of the experimental results, we chose a modified modeling method that changed the tap water drinking time in the last cycle to 7 days according to the literature and evaluated the therapeutic effect of IR on UC. The results showed that IR ameliorated the general symptoms of UC mice in a dose-dependent manner, including bloody stools, weight loss, colon shortening, and the pathological damage of the colon. Although balsalazide as the positive control drug can also relieve the symptoms of UC, in the treatment of chronic UC, the liver coefficient of mice increased after long-term administration, which suggests that long-term use of balsalazide may lead to side effects on the liver.

Cytokines participate in multiple biological processes of the organism, including immune and inflammatory responses (Feghali and Wright, 1997). Overactivity of pro-inflammatory factors such as IL-1β, IL-6, and TNF-α play important roles in inducing and maintaining colitis intestinal inflammation. The NF-κB pathway is activated in IBD patients, accompanied by the upregulation of inflammatory factors such as IL-1β, TNF-α, and IL-6 (Park and Hong, 2016). As a downstream target of the IL-6 signaling pathway, STAT3 is considered an important pathway leading to UC (Hirano, 2021). The IL-6-mediated STAT3 signaling pathway is the main target for the treatment of colorectal cancer and IBD (Akanda et al., 2018). We detected the mRNA levels of cytokines including IL-1β, IL-6, TNF-α, and IL-6/STAT3 pathway-related protein expression, and found that IR treatment could reduce the inflammatory response in mice by reducing the production of cytokines and inhibiting the IL-6/STAT3 signal pathway. A moderate inflammatory response is conducive to the body’s self-protection, but an excessive inflammatory response can lead to cell apoptosis and oxidative stress. Hagiwara et al. (2002) confirmed that apoptosis is the main reason for intestinal epithelial cell loss in UC patients, and excessive apoptosis will counteract epithelial defense and aggravate the disease in patients with active UC. Pro-inflammatory factors can cause oxidative stress by promoting the production of reactive oxygen species by immune cells. The continuous accumulation of oxidative stress weakens the immune system and further aggravates UC (Jeon et al., 2020). Our study showed that IR can reduce apoptosis and promote the regeneration of colonic epithelial cells, reduce the expression of Keap1 protein, and increase the expression of HO-1 and Nrf2 proteins at the same time, thereby enhancing the body’s antioxidant capacity, further preventing damage to the colon of UC mice.

Tight junctions and adherent junctions are important components of the intestinal mucosal barrier. Proteins of the occludin, claudin, and ZO families form a barrier at the top of the adjacent epithelial cell membrane to prevent the paracellular transport of intercellular molecules (Mehandru and Colombel, 2021). Adhesive proteins such as E-cadherin and β-catenin located under the basolateral of tight junctions interact with tight proteins and form adhesions between adjacent epithelial cells to close the intestinal barrier. The homeostasis of the intestinal epithelial barrier depends on the dynamic balance between apoptosis and proliferation, and its damage is considered an important pathogenic factor leading to IBD (Patankar and Becker, 2020). We found that the intestinal mucosal barrier of UC mice was severely damaged, the acidic mucous layer was destroyed, and LPS extravasated into the blood. IR repaired the damaged acidic mucous layer and upregulated the protein expression of occludin, claudin-1, and ZO-1 to promote the recovery of the intestinal epithelial barrier.

OSM is a multifunctional cytokine, belonging to the IL-6 cytokine family, which can be secreted by T cells, macrophages, and neutrophils. OSM mainly activates the corresponding signaling pathway by combining with its receptor OSMR and plays an important role in inflammation, cell growth, and hematopoiesis (Kalla et al., 2021). West et al. (2017) reported that the overexpression of cytokines OSM and OSMR in the intestinal tissues of IBD patients is positively correlated with histopathology disease severity, and can be used to predict the response of IBD patients to anti-TNF-α agents. Verstockt et al., 2021 considered that OSM can be used as a diagnostic and postoperative recovery biomarker in the tissues and serum of IBD patients. In a previous study, through transcriptome sequencing, we found that OSM and OSMR are crucial in the pathogenesis of UC and treatment with FYC (Li Y. et al., 2021). The OSM and OSMR proteins in the colon of UC mice were significantly increased, and FYC could ameliorate UC by reducing the expression of OSM and OSMR (Li X. X. et al., 2021). Because IR is an important prescription drug in FYC and plays an important role in the treatment of UC with FYC, and in the prescription of FYC lacking IR, the curative effect of prescription in the treatment of UC is weakened. Therefore, we studied the modulating effect of IR on OSM/OSMR. We detected the protein expression of OSM and OSMR in the colon of mice and found that OSM and OSMR proteins levels were significantly higher than those in normal mice, which were reduced by IR treatment. The protein expression of OSM and OSMR was increased in Caco2 cells stimulated with LPS, and IR pretreatment inhibited the increase of OSM and OSMR of Caco2 cells caused by LPS, thereby reducing the cellular inflammatory response.

Thus, IR exerts therapeutic effects on UC by regulating the OSM/OSMR signaling pathway to reduce colonic inflammation and colonic epithelial cell apoptosis, thereby improving antioxidant capacity and protecting the intestinal mucosal barrier in mice.

## Data Availability

The original contributions presented in the study are included in the article/Supplementary Material, further inquiries can be directed to the corresponding authors.
