# Supplementary material for: Ilex rotunda Thunb Protects Against Dextran Sulfate Sodium-Induced Ulcerative Colitis in Mice by Restoring the Intestinal Mucosal Barrier and Modulating the Oncostatin M/Oncostatin M Receptor Pathway
Source: Front Pharmacol. 2022 May 13;13:819826. doi: 10.3389/fphar.2022.819826 (PMC9140055; doi:10.3389/fphar.2022.819826)
Supplement: Supplementary file 1 [file DataSheet1.docx]

**Supplementary figures**

**
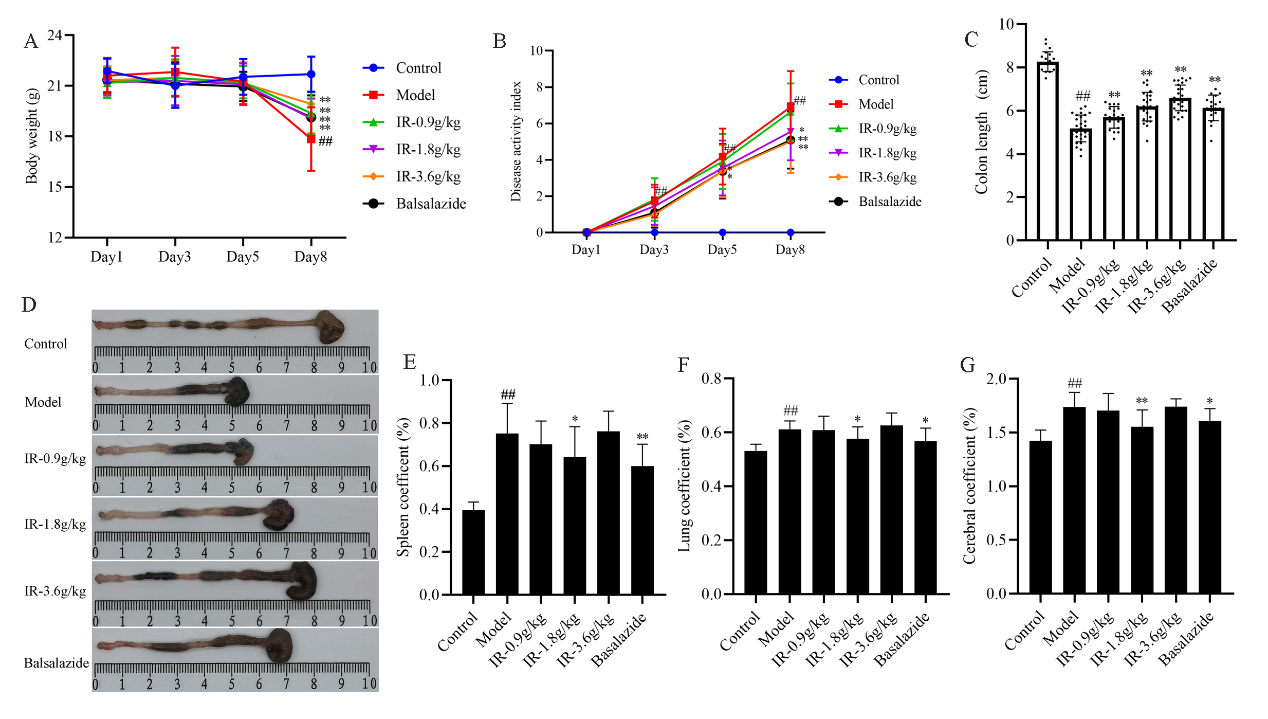
**

**Supplementary Figure. 1** IR ameliorated the pathological symptoms in acute UC mice. **(A)** The body weight from day 1 to day 8 in each group. **(B)** Disease activity index of each group. **(C)** colon length of each group at day 8. **(D)** Representative colon images at day 8. **(E)** Spleen coefficient of mice in each group. **(F)** Lung coefficient. **(G)** Cerebral coefficient. All data were compared using one-way ANOVA, and *p*-values reﬂected differences between experimental groups.

**
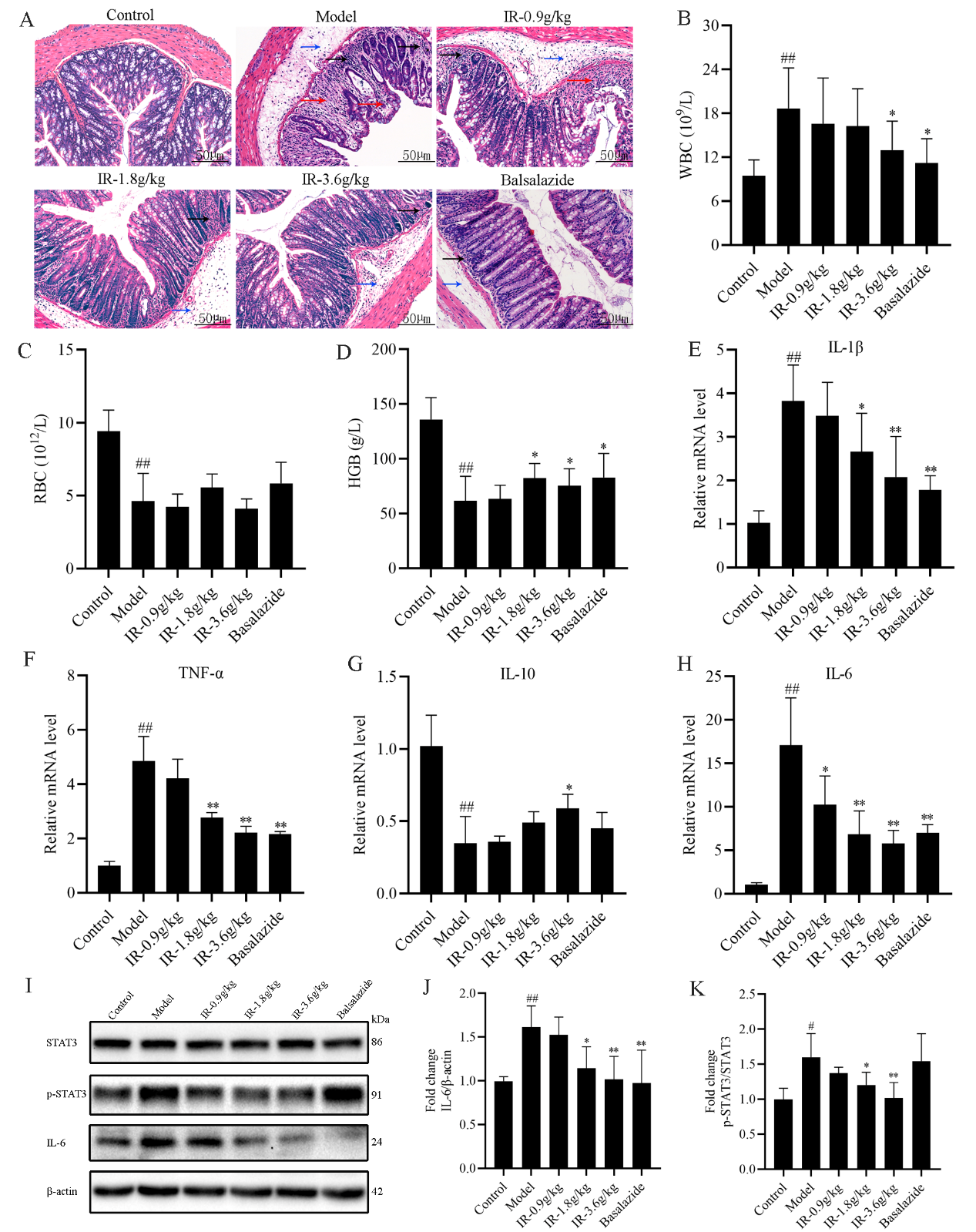
**

**Supplementary Figure. 2** IR suppress inflammation of acute UC mice. **(A)** Representative H&E staining colon tissue of mice (day 8). **(B)** The number of WBC in each group of mice. **(C)** The number of RBC in each group of mice. **(D)** The concentration of HGB in each group of mice. **(E-H)** The mRNA levels of IL-1β, TNF-α, IL-10, and IL-6 from the colon of mice. **(I)** The protein levels of STAT3, p-STAT3, and IL-6 in colon detected by western blotting. **(J)** The gray intensity analysis of IL-6. **(K)** The gray intensity analysis of p-STAT3/STAT3. All data were compared using one-way ANOVA, and *p*-values reﬂected differences between experimental groups.

**
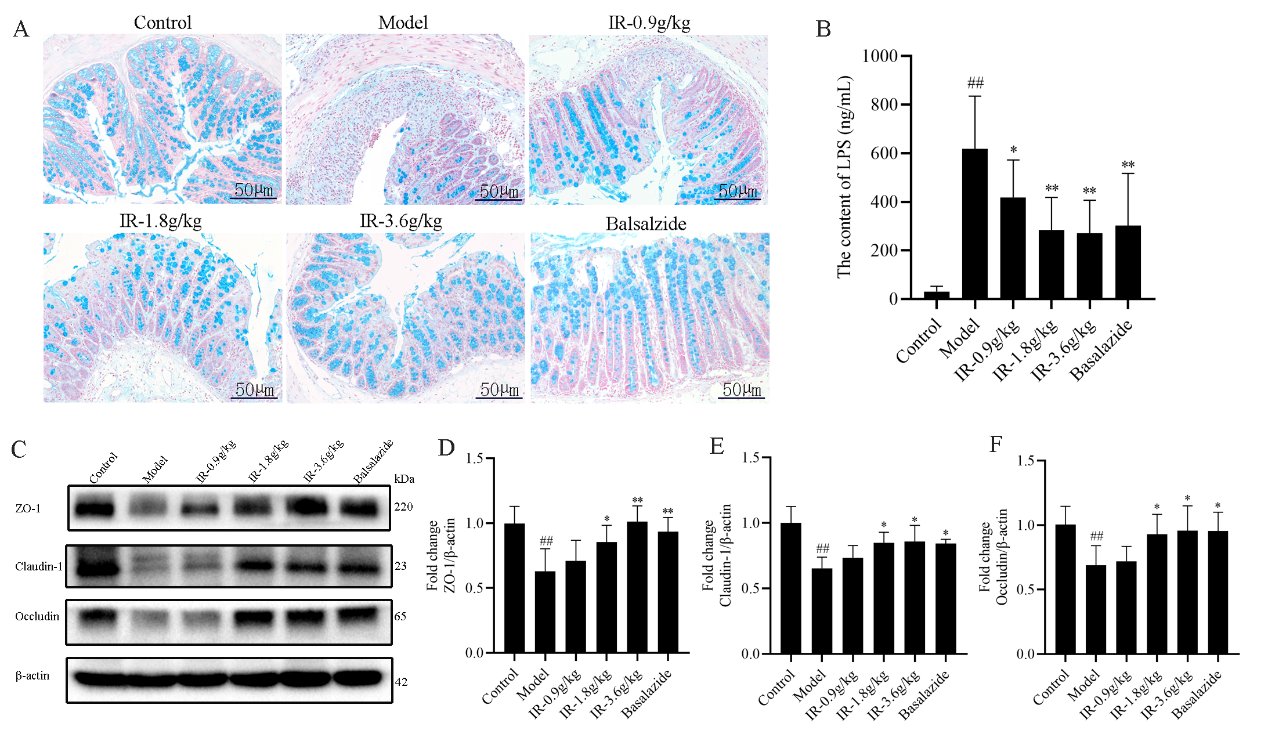
**

**Supplementary Figure. 3** IR alleviates intestinal mucosal barrier damage of acute UC mice. **(A)** Detection of acidic mucus layer in colon of mice by alcian blue staining (n=6). **(B)** The LPS content in serum of mice (n=6). **(C)** The protein levels of ZO-1, claudin-1, and occludin in colon detected by western blotting. **(D-F)** The gray intensity analysis of ZO-1, claudin-1, and occludin proteins, respectively (n=3). All data were compared using one-way ANOVA, and *p*-values reﬂected differences between experimental groups.

**Supplementary table**

Table 1 The primers sequence information for qRT-PCR

| Gene | Sequence |
| --- | --- |
| IL-1β | F: CTGCAGCTGGAGAGTGTGGA |
|  | R: TGCCCTGGGGAAGGCATTAG |
| IL-6 | F: TGAACAACGATGATGCACTTGCAGA |
|  | R: CTGTGACTCCAGCTTATCTCTTGGT |
| TNF-α | F: ATTCGAGTGACAAGCCTGTAGCC |
|  | R: CTCCACTTGGTGGTTTGCTACGA |
| β-actin | F: GACGGCCAGGTCATCACTATTG |
|  | R: CCACAGGATTCCATACCCAAGA |
